# The Age-Related Changes in Cartilage and Osteoarthritis

**DOI:** 10.1155/2013/916530

**Published:** 2013-07-22

**Authors:** YongPing Li, XiaoChun Wei, JingMing Zhou, Lei Wei

**Affiliations:** ^1^Department of Orthopaedics, The Second Hospital of Shanxi Medical University, 382 Wuyi Road, Taiyuan, Shanxi 030001, China; ^2^Department of Orthopaedics, The Warren Alpert Medical School of Brown University/Rhode Island Hospital, 1 Hoppin Street, Providence, RI 02903, USA

## Abstract

Osteoarthritis (OA) is closely associated with aging, but its underlying mechanism is unclear. Recent publications were reviewed to elucidate the connection between aging and OA. With increasing OA incidence, more senior people are facing heavy financial and social burdens. Age-related OA pathogenesis is not well understood. Recently, it has been realized that age-related changes in other tissues besides articular cartilage may also contribute to OA development. Many factors including senescence-related secretory phenotypes, chondrocytes' low reactivity to growth factors, mitochondrial dysfunction and oxidative stress, and abnormal accumulation of advanced glycation end products (AGEs) may all play key roles in the pathogenesis of age-related OA. Lately, epigenetic regulation of gene expression was recognized for its impact on age-related OA pathogenesis. Up to now, few studies have been reported about the role of miRNA and long-noncoding RNA (lncRNA) in age-related OA. Research focusing on this area may provide valuable insights into OA pathogenesis. OA-induced financial and social burdens have become an increasingly severe threat to older population. Age-related changes in noncartilage tissue should be incorporated in the understanding of OA development. Growing attention on oxidative stress and epigenetics will provide more important clues for the better understanding of the age-related OA.

## 1. Introduction

Half of the world's population, aged 65 and older, suffers from OA [1]. Many studies showed that increased age is the most prominent risk factor for the initiation and progression of primary OA in typically affected joints including interphalangeal, hips, knees, and intervertebral. The rare cases of OA diagnosed in young individuals, under the age of 25–30 years old, are mostly due to mutations in matrix genes that cause significant structural abnormalities and/or joint deformities [2–4]. 

To this day, the mechanism of OA has not been fully clarified. Current hypotheses include the classic “wear and tear” theory [2, 4], senescence-related secretory phenotype [5], chondrocytes' low reactivity to growth factors [6], mitochondrial dysfunction and oxidative stress [7], and abnormal accumulation of advanced glycation end products (AGEs) [8]. The cumulative effect of mechanical load over the years may cause “wear and tear” clinically and cartilage breakdown pathologically [2]. Hence, OA is regarded as a naturally occurring irreversible phenomenon, rather than a specific, potentially treatable disease. However, OA is not inevitable for all senior adults (age 60+). It has become increasingly clear that OA is not a purely mechanical problem [9]. Age-related changes in cartilage extracellular matrix proteins such as collagen or proteoglycans can induce nonenzymatic collagen cross-linking and shorten aggrecan molecules [9]. Excessive collagen cross-linking affects the biomechanical properties of cartilage, causes increased stiffness, and makes cartilage more brittle [10] and susceptible to fatigue failure [11]. Shortening and degradation of aggrecan leads to significant loss of sugar side chains and water-binding ability [12]. In addition, increased levels of AGEs are associated with a decline in anabolic activity [13]. These findings suggest that age-related changes in matrix may play a role in the development of OA.

Lately, it has been gradually realized that age-related changes occurring in tissues besides articular cartilage may contribute to the development of OA [9]. Because articular cartilage lacks nerve supply, knee pain could be attributed to OA-related changes of the joint tissues, such as the synovium, bone (including osteophytes), joint capsule, ligaments, and menisci [14]. These tissues could play important roles in the early stages of OA [14]. Thus, OA is considered a “whole joint disease” [9]. Although it raises the complexity of OA, this concept improved our understanding of the disease as well as indicated potential new treatment strategies [9].

This review focuses on recent studies that shed light on the connection between aging changes in cells and tissues and the propensity for OA occurrence in older adults. 

## 2. Epidemiology

OA is the most common joint disorder in the world and one of the most common sources of pain and disability in the elderly [15, 16]. The incidence of OA is predicted to increase as the senior population grows, placing a significant financial burden on healthcare providers and governments [17]. OA affects at least 27 million Americans and is the leading cause of disability in the United States [17]. Compared to only 7.6% of those 18–44 years of age and 29.8% of those 45–64 years of age, 50% of individuals older than 65 years are diagnosed with this disease [17]. OA affects one in six adults, and by 2030 it is estimated that 20% of people in Europe and the United States will suffer from OA [18]. The senior population is growing rapidly in many Asian countries [19]. It is estimated that the 65+ population in Asia will more than double in the next two decades, increasing from 6.8% in 2008 to 16.2% in 2040. In most of the developed world demographic change is a gradual progress following the steady socioeconomic growth over several decades. In contrast, this change is compressed into 2-3 decades in many Asian countries. For example, during the period between 2008 and 2040, it is estimated that the 65+ population will increase by 316% in Singapore, 274% in India, 269% in Malaysia, 261% in Bangladesh, and 256% in the Philippines. In 2008, Japan had the world's oldest population (people 65+ represented 21.6% of whole population), and both China and India were ranked top two for the size of 65+ population (106 and 60 million, resp.) [16]. The high prevalence and heavy impact on working capacity make OA a major social issue [20]. Therefore, healthcare and socioeconomics need to put a high priority to the prevention and treatment of OA [18].

OA in humans usually becomes symptomatic after age 50, which is also when the radiographic changes of OA become more common [4]. Although radiographic signs of OA such as osteophytes and joint space narrowing do not always correlate well with symptoms, epidemiologic studies of large cohorts commonly depend on radiographs to define OA [4]. Goekoop et al. examined a cohort of 90-year olds living in the city of Leiden in Netherlands and found that only 16% of people in that age were free of radiographic OA [21]. 

In the Johnson Country OA cohort [22], the prevalence of radiographic knee OA rose from 26.2% in the 55–64 age group to nearly 50% in the 75+ age group, and the prevalence of symptomatic knee OA likewise increased from 16.3% to 32.8% between these age groups. Symptomatic hip OA in this cohort was reported as 5.9% in the 45–54 age group compared with 17% in the 75+ age group. In the Johnston County OA Project, African American men had a higher prevalence of radiographic hip OA than Caucasian men (32.2% versus 23.8%), whereas no difference was found between African American and Caucasian women (40.3% versus 39.4%) [23]. Individual radiographic features in hip and knee were also noted to differ between two ethnic groups [24, 25]. In the Beijing OA Study, hand and hip OA were less prevalent among Chinese than Caucasians (age-standardized prevalence 44.5%–47% versus 75.2%–85% and 0.8% versus 3.8–4.5%, resp.), but knee OA was more prevalent among Chinese women than Caucasian women (46.6% versus 34.8%) [16, 18, 26].

The heritability of OA is estimated to be 40% to 65% and is higher for hand and hip OA than for knee OA [27–29]. So far, 3 loci have been associated to OA at genome-wide significant levels [30–32], that is, GDF5, which encodes the growth differentiation factor 5 (a bone morphogenetic protein expressed in skeletal and articular structures), chromosome 7q22, and MCF2L (MCF.2 cell line derived transforming sequence-like). Pain severity in OA may also have genetic contributions. A functional polymorphism (Val158Met) in the COMT gene, which was previously correlated with pain sensitivity in other clinical conditions, was associated with hip OA-related pain in a cohort study [33]. TRPV1 and the PACE4 gene Pcsk6 were associated with pain in knee OA in two separate meta-analyses [34, 35].

## 3. Chondrocyte Changes

The primary function of chondrocytes is to maintain cartilage homeostasis, in part through the production of extracellular matrix components. With age, chondrocytes exhibit features similar to senescent phenotypes, including telomere shortening and increased senescence-associated *β*-galactosidase activity [5, 36]. These age-related changes impair the ability of chondrocytes to maintain the surrounding extracellular matrix. Accordingly, in aged chondrocytes, synthetic activity is decreased and proteoglycans are smaller and more irregular [37, 38].

A reduction in the number of chondrocytes was observed in normal articular cartilage during aging, comparing with a greater loss of chondrocytes in OA cartilage, but the extent of cell death is debatable [39–41]. A study showed 30% drop in cell density in human hip joint cartilage between the ages of 30 and 70 [42]. However, a study on human knees found less than 5% cell loss during aging [43]. Loss of the chondrocytes can be attributed to increased chondrocyte death and/or apoptosis. Although many studies reported apoptotic chondrocytes in OA cartilage [41], few have examined apoptosis in cartilage with normal aging, except for one study on rat cartilage [44]. There is evidence showing [45] that HMGB2, a high-mobility group box (HMGB) protein that may be important for chondrocyte survival, regulates gene transcription through chromatin organization. HMGB2 is mainly expressed in chondrocytes in the superficial zone of articular cartilage, and HMGB2 levels drop during aging [45]. Moreover, the decline in HMGB2 levels was associated with increased chondrocyte death, and HMGB2-deleted mice developed premature OA [45]. Levels of reactive oxygen species (ROS) are increased in cartilage during aging, and chondrocytes from older adults are more susceptible to ROS-mediated cell death [46].

The synthetic activity of chondrocytes is regulated by anabolic growth factors [47]. During aging, chondrocytes exhibit reduced responsiveness to growth factors, such as insulin-like growth factor-1 (IGF-1) [6, 48, 49], osteogenic protein-1 (OP-1) or bone morphogenic protein-7 [50], and transforming growth factor-*β* (TGF-*β*) [51, 52]. For example, TGF-*β* stimulates proteoglycan synthesis in young animals, but this ability is impaired in old mice [51, 53]. It is hypothesized that age-related alterations in the TGF-*β* signaling pathway trigger chondrocytes to leave their normally quiescent state into an autolytic phenotype, leading to degradation of cartilage extracellular matrix [54]. Reduced anabolic response to IGF-I was also noted in chondrocytes isolated from OA cartilage [48, 55]. These findings suggest that age-related decline in anabolic activity could tip the balance towards increased catabolism and play a key role in increasing cartilage susceptibility to OA. 

Age-related changes inside chondrocytes including cellular senescence and reduced responsiveness to growth factors and extracellular factors affecting chondrocyte aging such as AGE accumulation and oxidative stress may work together to disrupt cartilage homeostasis. These changes will make the cartilage matrix more vulnerable to damage and lead to the onset of OA. The onset of OA is characterized by increased cell proliferation, which leads to formation of chondrocyte clusters and increased synthesis of irregular matrix components such as collagens and proteoglycans [56–58]. With OA progression, excessive catabolic activity causes imbalance of cartilage homeostasis and cartilage matrix breakdown. These catabolic events are largely mediated by proinflammatory cytokines and mediators, for example, matrix metalloproteinases (MMPs), and a disintegrin and metalloproteinase with thrombospondin motifs (ADAMTS) [59]. Notably, many characteristics of an aged chondrocyte parallel changes observed in early OA, which might explain why age is highly correlated to OA [60].

AGEs are produced through a nonenzymatic reaction between reducing sugars and free amino groups of proteins, lipids, or nucleic acids [61]. Excessive levels of AGEs in the body are pathogenic, resulting in elevated oxidative stress and inflammation [62]. In chondrocytes, AGEs can increase the production of inflammatory cytokine tumor necrosis factor-*α* (TNF-*α*), inflammatory mediators prostaglandin E2, and nitric oxide. It can also suppress the production of type II collagen and stimulate the expression of degradative enzyme MMPs and ADAMTS [8, 63, 64]. AGEs accumulation also has adverse effects on the cartilage extracellular matrix. AGEs increase collagen cross-linking, which enhances tissue stiffness, making cartilage more brittle and susceptible to mechanical failure [10, 11, 65]. Although not reported in chondrocytes, AGEs also induce ROS generation in murine hepatic stellate cells and bone marrow mesenchymal stem cells [66, 67]. 

ROS play important roles in many physiological processes and can potentially cause oxidative damages to proteins, lipids, and DNA [68]. Human articular chondrocytes actively produce ROS, and increased levels of ROS were observed in articular cartilage of old rats compared to young rats [69–72]. Furthermore, cartilage of old rats exhibited a significant drop in antioxidant catalase activity [72]. This redox imbalance may be caused by an age-related decline in the activity and number of mitochondria, which play critical roles in protecting cells from ROS damage [7]. The consequence of increased oxidative stress is DNA damage and telomere shortening, leading to reduced matrix production, chondrocyte senescence, and apoptosis [73–75]. Increased ROS also upregulate proinflammatory cytokines and MMPs, factors that mediate cartilage degradation [76]. 

Mitochondria is a major source of ROS in the cell, and mitochondrial dysfunction is thought to play a key role in age-related diseases including OA. Evidence has been shown that mitochondrial DNA damage in OA is promoted by inflammatory cytokines such as IL-1*β* and TNF-*α* and contributes to chondrocyte death [76]. Mechanical injury to cartilage, such as articular cartilage crushing, shearing force injury, would result in elevated ROS generation in mitochondria and promotes chondrocyte death [77]. Subchondral bone softening, which occurs during age-related osteoporosis [78], is predicted to alter the biomechanics of the tibiofemoral joint by increasing the maximum tensile strains in cartilage and the magnitudes of joint contact pressure [79]. In addition, due to the aged less activity, the declined quadriceps strength may be another factor responsible for altered joint loading patterns as a consequence of joint laxity [80]. Nonphysiological load or less mechanical load exerted on chondrocytes would induce catabolic signaling and cartilage tissue breakdown [59].

**Figure 1:**
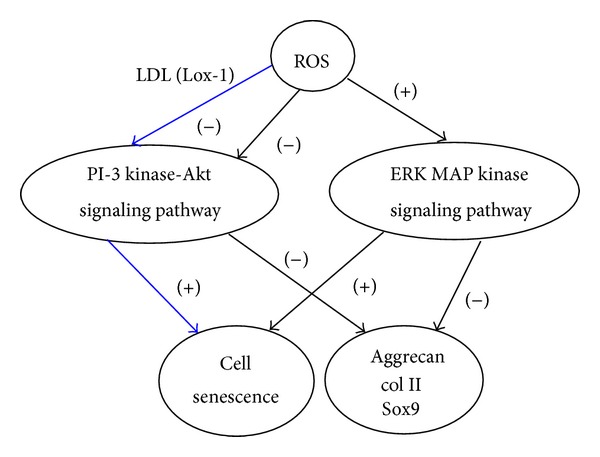
Schematic diagram showing the key role of ROS in age-related chondrocyte changes. Excessive levels of ROS inhibited matrix synthesis (aggrecan, type II collagen) by suppressing the IRS-1-PI-3 kinase-Akt signaling pathway or by activating the ERK MAPK signaling pathway. Sustained activation of ERK can induce cell senescence. In addition, extracellular ROS could also contribute to the inhibition of the Akt pathway through oxidized low-density lipoprotein (LDL). The binding of oxidized LDL to cell surface receptor LOX-1 was found to induce chondrocyte senescence (blue arrow).

ROS could play a key role in age-related chondrocyte changes in several signaling pathways [81]. Excessive levels of ROS were found to inhibit activation of the IRS-1-PI-3 kinase-Akt signaling pathway, which normally promotes matrix synthesis [81] (Figure 1). Meanwhile, ROS activates the ERK MAP kinase which suppresses the expression of chondrocyte aggrecan, type II collagen, and Sox-9 [77] (Figure 1). Sustained activation of ERK can induce cell senescence. A study [82] using rat chondrosarcoma cells demonstrated that sustained ERK activation mediated by FGFR3 promoted the expression of the senescent phenotype markers. Extracellular ROS could also contribute to inhibition of the Akt pathway through oxidized low-density lipoprotein (LDL). The binding of oxidized LDL to cell surface receptor LOX-1 was found to induce chondrocyte senescence, possibly by inhibiting Akt phosphorylation upon IGF-1 stimulation [83] (Figure 1). Oxidative stress induced by oxidized LDL is also associated to promotion of hypertrophic chondrocyte phenotype in OA cartilage [84].

A study [85] on the expression of the superoxide dismutase (SOD) family of antioxidants demonstrated decreased expression of all three SOD isoforms (copper/zinc (Cu/Zn)-SOD, manganese (Mn)-SOD, and extracellular (EC)-SOD), at the transcriptional level. Decreased expression of mitochondrial SOD (SOD2) was associated with increased methylation inSOD2 promoter region suggesting that epigenetic regulation may be involved in inhibition of the expression of this antioxidant gene [85]. A more recent study reported that people in Spain with mitochondrial DNA haplotype J, which is associated with a lower risk of OA, have lower serum levels of MMP-13 when compared to those with haplotype H, who have higher serum levels of MMP-3 [86]. It is possible that the different haplotypes are characterized by different ROS generation and perhaps different amount of mitochondrial DNA damage, although this is not fully established. Interestingly, a study examining a murine model of premature aging that exhibits increased nuclear DNA damage due to deficiency of a repair enzyme found a significant increase in age-related bone loss but not in cartilage damage [87].

## 4. The Changes in Cartilage Matrix

Age-related changes not only occur in chondrocytes but also in cartilage matrix, thereby contributing to OA development. MRI studies showed that knee cartilage thins during aging, particularly on the femoral side of the joint [88] and in patellae [89], suggesting a gradual loss of cartilage matrix with age. This could be due to loss of chondrocytes and reduced growth factor activity, but also to something as simple as reduced water content. Type II collagen, the most abundant matrix protein in cartilage, has a half-life over 100 years [90]. Excessive collagen cross-linking increases stiffness and brittleness [10], thereby increasing susceptibility to fatigue failure [11]. Increased levels of AGEs in cartilage are correlated with declined anabolic activity [13]. Being the second most abundant cartilage matrix protein, aggrecan is a large “aggregating” proteoglycan consisting of a core protein and numerous highly sulfated glycosaminoglycan chains that are covalently attached [91]. Because of the hydrophilic nature of aggrecan's negatively charged sulfates, articular cartilage has about 70–80% water content and is very resilient. Age-related changes in size, structure, and sulfation of aggrecan [12, 37, 91, 92] affect cartilage resiliency and hydration [93]. When aggrecan is degraded, a fragment containing the binding region for hyaluronic acid is left behind and appears to accumulate in cartilage with age due to a low turnover rate with an estimated aggrecan half-life of 25 years in cartilage [94].

The balance of anabolism to catabolism is regulated by the fine-tuning of the specific genes in certain signaling pathways. Studies using transgenic and knockout mice continue to provide information on specific genes that may play a role in OA progress [95]. FGFR3 knockout mice [96] were found to develop more severe OA with age than FGFR1 knockout mice [97]. Together, these results suggest an anabolic/joint protective function of FRGFR3 and a catabolic/joint destructive function for FGFR1. 

**Figure 2:**
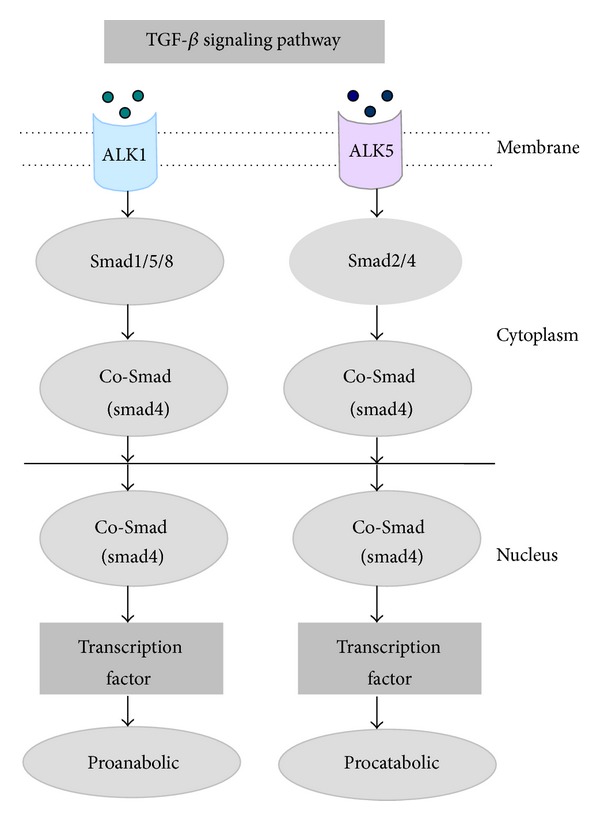
Schematic diagram showing that cartilage matrix homeostasis is adjusted dynamically by TGF-*β* signaling pathway. Activated TGF-*β* binds to the TGF-*β* type I receptor ALK1, resulting in phosphorylation of Smad1, Smad5, or Smad8, which form a complex with the co-Smad Smad4 and translocate to the nucleus to promote cartilage matrix anabolism by modifying gene expression. Meanwhile, the activated TGF-*β* type II receptor ALK5 results in phosphorylation of Smad2 or Smad3 which form a complex with the co-Smad Smad4 and translocate to the nucleus to promote cartilage matrix catabolism by modifying gene expression.

Transforming growth factor-*β* (TGF-*β*) is secreted in an inactive form and requires activation before binding to its receptor [98]. Activated TGF-*β* binds to the TGF-*β* type II receptor and forms a complex that recruits the TGF-*β* type I receptor, ALK5. However, TGF-*β* is also able to signal via the alternative TGF-*β* type I receptor ALK1 in chondrocytes [99] (Figure 2). In endothelial cells as well as chondrocytes, activation of ALK5 is followed by Smad2 or Smad3 phosphorylation, while ALK1 activation results in phosphorylation of Smad1, Smad5, or Smad8 [54, 99–101] (Figure 2). The activated Smads form a complex with the co-Smad Smad4 and translocate to the nucleus to modify gene expression. Interestingly, signaling via either ALK5 or ALK1 can turn the response of cells to TGF-*β* stimulation in opposite directions [54, 102] (Figure 2). For example, in endothelial cells ALK5 inhibits migration, whereas ALK1 stimulates migration and proliferation [103]. The Smad pathway appears to be the most important for TGF-*β* signaling but is not the only option. Mitogen-activated protein kinase, Rho-like GTPase, and phosphatidylinositol-3-kinase pathways are involved in TGF-*β* signaling [104]. Activation of TGF-*β* activated kinase 1 (TAK1) happens independently of ALK5 kinase activity and induces P38 and JNK signaling [105]. Studies on activin receptor-like kinases (ALKs) activated by TGF-*β* showed that ALK5 activation is proanabolic, and ALK1 activation is procatabolic [106] (Figure 2). During aging and in OA, the ratio of ALK1 to ALK5 is increased to promote OA development, and the ratio of FGFR1 to FGFR3 may change in a similar way [107].

In our previous study, we demonstrated the loss of TGF-*β* type I receptor ALK5 and phosphorylation of Smad2/3 in murine articular cartilage during aging [108], but the expression of total Smad2 was not altered by TGF-*β*. Moreover, in two experimental models of OA—the DMM (meniscus destabilization) model and the STR/ORT mice (spontaneous OA model)—development of the disease was correlated with decreased ALK5 expression. Expression of the alternative TGF-*β* receptor ALK1 did not decrease to a similar extent as ALK5 [109]. STR/ORT mice develop OA starting at the medial tibia from an age of 2 to 3 months. The ALK1/ALK5 ratio was 5 on the medial tibia at the age of 3 months and was 18 in 1-year-old animals. The lateral tibia showed increased ratio from 1 to 5 over the same period of time. Clearly increased ALK1/ALK5 ratios in chondrocytes are associated with aging and OA development [110].

## 5. Chondrocyte Senescence

Chondrocytes are very unique cells that easily develop into age-related changes with aging. The chondrocytes present in the articular cartilage of an 80-year old are likely to be the very same cells as those present in a 25-year old. There is little to no cell division or cell death in normal adult articular chondrocytes [43], and there seems to be no ready supply of progenitor cells to replace dead chondrocytes if cell death does occur. The articular chondrocytes which underwent more cell divisions exhibit telomere shortening [111]. Aging itself is not associated with chondrocyte proliferation, but rather with loss of normal mitogenic response of isolated chondrocytes to growth factor stimulation [112]. 

There are two types of senescence: intrinsic and extrinsic. The classic replicative senescence or “intrinsic senescence” is attributed to shortened telomeres accompanied by telomere dysfunction [113]. Evidence of telomere shortening in chondrocytes from older adults has been reported [114]. However, the senescence in articular cartilage seems more relevant to the “extrinsic senescence” or stress-induced senescence, which occurs in response to telomere-damaging stimuli, including oxidative damage, activated oncogenes, and inflammation [113, 115] and is a much more likely mechanism for senescence in cartilage [116].

Accumulation of cells exhibiting the senescent secretory phenotype contributes to tissue aging [115, 117]. This phenotype is characterized by increased production of cytokines including IL-6 and IL-1, matrix metalloproteinases, and growth factors such as EGF with some features in common with the OA chondrocyte phenotype. Studies have shown increased expression of MMP-3 and MMP-13 in aged cartilage [118, 119] as well as age-related accumulation of collagen neoepitopes representing denatured or cleaved collagen [120, 121]. It was shown that increased MMPs mediate cartilage matrix damage during aging, and collagenases and cathepsin K were also implicated in this process recently [122].

Other mediators of cellular senescence include TRF (telomeric repeat binding factor), XRCC5 (X-ray repair complementing defective repair in Chinese hamster cells 5), and SIRT1 (sirtuin 1). TRF1 and TRF2 are telomeric proteins that function to form and maintain telomere structure [123, 124]. XRCC5 is involved in repairing DNA double-strand breaks [125]. SIRT1 is a negative regulator of p53 and prevents growth arrest, senescence, and apoptosis [126]. Oxidative stress in human chondrocytes induces senescence and accelerates telomere shortening [127]. After acute oxidative insult, TRF1, TRF2, XRCC5, and SIRT1 are upregulated in the early passages of human chondrocytes but induced to a less extent in late passages of chondrocytes [127]. This finding suggests that TRF proteins, XRCC5, and SIRT1 help young chondrocytes to cope with oxidative stress by preventing DNA damage accumulation and telomere shortening. Consistently, aged chondrocytes with lower induction levels of these regulatory proteins have a reduced tolerance to oxidative challenge, and accumulation of DNA damage may trigger chondrocyte senescence. Membrane protein caveolin-1 is also involved in senescence. Expression of caveolin proteins is increased in tissues of old rats [128], and overexpression of caveolin-1 leads to a senescent phenotype, likely through the p53/p21 pathway [116]. In addition, angiogenic growth factor (AGF) treatment in human chondrocytes downregulated interleukin-1*β*- (IL-1*β*-) induced caveolin-1 expression and prevented chondrocyte replicative lifespan shortening. Inhibition of p42/p44 mitogen-activated protein kinase (MAPK) and phosphoinositide 3-kinase (PI3K) abolished the effect of AGF on caveolin-1, suggesting that the AGF-mediated inhibition of IL-1*β*-induced chondrocyte aging is regulated, at least in part, by p42/44 MAPK and PI3K [129]. 

**Figure 3:**
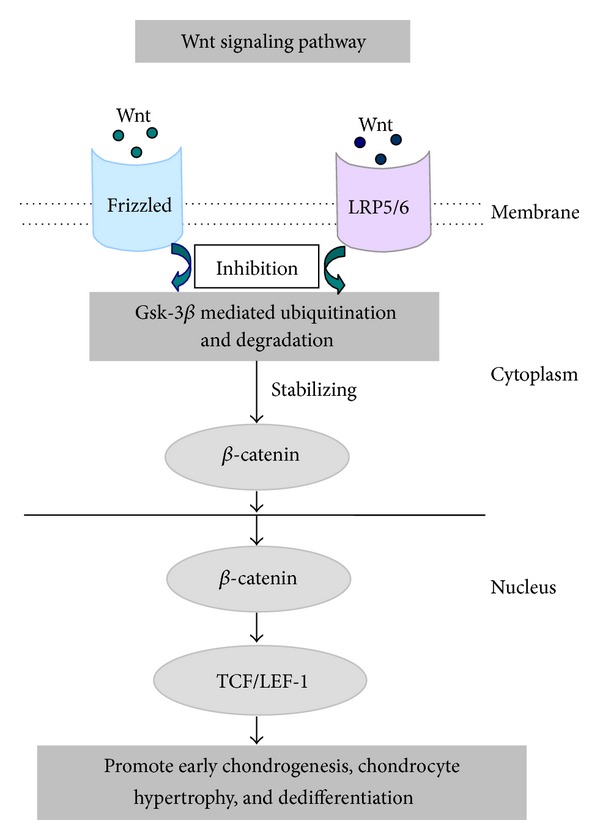
Schematic diagram showing the role of Wnt signaling in age-related changes of cartilage. Wnt could bind to Frizzled receptors and LRP5/6 coreceptors and lead to stabilization of *β*-catenin via inhibition of GSK-3*β* mediated ubiquitination and degradation. Then *β*-catenin translocated to the nucleus and bound to TCF/LEF-1 transcription factors, which can inhibit and promote early chondrogenesis as well as promote hypertrophy and chondrocyte dedifferentiation.

The Wnt family of secreted glycosylated proteins are linked to the development of a number of age-related pathologies such as osteoarthritis. Manipulation of Wnt signaling has the potential to impact both cellular survival and longevity; however, aberrant Wnt signaling can promote cell senescence [130]. Disruption of Wnt signaling has also been associated with altered joint formation, chondrogenesis, and OA [131–133]. Wnt signaling occurs through canonical (*β*-catenin dependent) and noncanonical (*β*-catenin independent) pathways. In canonical Wnt signaling, Wnt binding to Frizzled receptors and LRP5/6 coreceptors leads to stabilization of *β*-catenin via inhibition of GSK-3*β* mediated ubiquitination and degradation (Figure 3). *β*-catenin can then translocate to the nucleus and bind to TCF/LEF-1 transcription factors (Figure 3). OA-like changes occur in mice following both under- and overactivation of the Wnt pathway [134] (Figure 3). Canonical Wnt signaling has been reported to both inhibit and promote early chondrogenesis and to promote hypertrophy and chondrocyte dedifferentiation [135] (Figure 3). In contrast, the noncanonical pathway, through Wnt5a and Wnt5b, can promote chondrogenesis and inhibit hypertrophy [136]. This suggests that fine regulation of the Wnt pathway is essential for proper cartilage development and homeostasis. Recently, activation of the Wnt pathway was shown to inhibit IL-1-mediated MMP-13 expression in human chondrocytes. This was TCF/LEF independent and mediated through a direct interaction between NF-*κ*B and *β*-catenin, suggesting a potential protective function of Wnt in aging and OA [137]. Interestingly, HMGB2 and Wnt activity colocalize in the superficial zone of articular cartilage [138]. Age-related loss of HMGB2 and OA could also be associated with loss of Wnt activity in the superficial zone of articular cartilage, although Wnt activity is enhanced in other zones within articular cartilage, along with osteophytes and subchondral bone [138]. Understanding the impact and mechanisms underlying the imbalance of Wnt activity across the joint will provide insights into aging and OA-related cartilage degradation.

## 6. Epigenetics

There is a growing interest in the role epigenetics play in age-related conditions including OA. Epigenetic regulation of gene expression includes DNA methylation, histone acetylation and methylation, and micro-RNA (miRNA). Sirtuins are a family of NADt-dependent deacetylases that are linked to aging and more recently shown to be involved in OA through the regulation of cellular energy and metabolism [139]. The sirtuin SirT1 promotes chondrocyte survival and matrix gene expression. TNF-*α* cleaves and inactivates SirT1 and thereby contributes to reduced matrix gene expression [140]. Mice heterozygous for SirT1 (+/−) with significantly decreased SirT1 expression developed premature OA-like changes at 9 months of age, which may be due to increased chondrocyte apoptosis [141].

Recent evidence [142] showed that a site within the promoter region of MMP-13 was demethylated in OA chondrocytes. It not only can make the cAMP response element bind to the promoter region but also can upregulate MMP-13 expression. Histone methylation is implicated in the age-dependent expression of the nuclear factor of activated T cells, cytoplasmic, calcineurin-dependent 1 (NFATc1) that promotes cartilage homeostasis [143]. In one study [144], miR-199a-3p and miR-193b were upregulated with age, whereas miR-320c was downregulated. These two upregulated miRNAs were found to reduce collagen and aggrecan expression *in vitro*, suggesting that they are antianabolic and may be involved in the age-related decrease in matrix gene expression. The development of postgenomics enabled the extensive and intensive study of the role of long noncoding RNA (lncRNA) in gene regulation [144]. So far, few studies on microRNA and lncRNA have been reported in the field of age-related OA [145]. A growing interest in this field of research may provide valuable clues to elucidate the pathogenesis of age-related OA.

## 7. Conclusion

OA-induced financial and social burdens have become more and more severe with aging of the population. Age-related changes in cartilage have been identified as critical factors in OA development. However, the underlying molecular mechanisms are not completely clarified yet, even though some theories have been proposed. Critical factors and signaling pathways that may play important roles in age-related changes of OA cartilage need to be further investigated. In addition, the importance of lncRNA in gene regulation is now better understood, and the potential role of lncRNAs as biological markers in diagnosis and prognosis of clinical diseases has also been considered. Studies focusing on these topics will provide more important clues for better understanding of the age-related OA.
